# Supplementary material for: End Sequence Analysis Toolkit (ESAT) expands the extractable information from single-cell RNA-seq data
Source: Genome Res. 2016 Oct;26(10):1397–410. doi: 10.1101/gr.207902.116 (PMC5052061; doi:10.1101/gr.207902.116)
Supplement: Supplemental Material [file supp_gr.207902.116_Supp_Materials.tar.gz › ESAT-esat_v0.1/doc/html/classbroad_1_1pda_1_1seq_1_1clip_1_1_pileup-members.html]

ESAT: Member List


ESAT


- Main Page
- Related Pages
- Packages
- Classes
- Files

- Class List
- Class Index
- Class Hierarchy
- Class Members

All Classes Namespaces Files Functions Variables Enumerator Pages

- broad
- pda
- seq
- clip
- Pileup

broad.pda.seq.clip.Pileup Member List

This is the complete list of members for broad.pda.seq.clip.Pileup, including all inherited members.

|  |  |  |
| --- | --- | --- |
| getAnnotation() | broad.pda.seq.clip.Pileup |  |
| getFragmentEndsFromBamFile(Annotation region, boolean fullyContained, boolean fivePrimeEnd) | broad.pda.seq.clip.Pileup |  |
| getScanPvalFragmentEndsMaxPositionAllRegionsBam(boolean fullyContained, Map< String, Double > nucProbs, int numPermutations, boolean fivePrimeEnd) | broad.pda.seq.clip.Pileup |  |
| getScanPvalFragmentEndsMaxPositionBam(Annotation region, boolean fullyContained, Map< String, Double > nucProbs, int numPermutations, boolean fivePrimeEnd) | broad.pda.seq.clip.Pileup |  |
| getScanPvalsFragmentEndPileupsAllRegionsWig(boolean fullyContained, Map< String, Double > nucProbs, int numPermutations) | broad.pda.seq.clip.Pileup |  |
| getScanPvalsFragmentEndPileupsWig(Annotation region, boolean fullyContained, Map< String, Double > nucProbs, int numPermutations) | broad.pda.seq.clip.Pileup |  |
| getShuffledPositions(Annotation region, boolean fullyContained, Map< String, Double > nucProbs, int numPermutations) | broad.pda.seq.clip.Pileup |  |
| getShuffledPositionsAllRegions(boolean fullyContained, Map< String, Double > nucProbs, int numPermutations) | broad.pda.seq.clip.Pileup |  |
| main(String[] args) | broad.pda.seq.clip.Pileup | static |
| Pileup(String bamFile, String annotationBedFile, String subregionBedFile, String genomeFasta, Collection< Predicate< Alignment >> readFilters) | broad.pda.seq.clip.Pileup |  |
| Pileup(String bamFile, String fragmentEndWig, String annotationBedFile, String subregionBedFile, String genomeFasta, String regionOverlapperCountFile, String peakRegionBedFile, Collection< Predicate< Alignment >> readFilters) | broad.pda.seq.clip.Pileup |  |
| writeScanPvalsFragmentEndPileupsAllRegionsWig(String outTable, String outBed, double pvalCutoffForBed, boolean fullyContained, Map< String, Double > nucProbs, int numPermutations) | broad.pda.seq.clip.Pileup |  |


---

Generated on Fri Aug 15 2014 15:11:01 for ESAT by  

 1.8.7
